# 3-[Hy­droxy(3-meth­oxy­phen­yl)methyl­idene]-2-(2-oxo-2-phenyl­eth­yl)-3,4-dihydro-2*H*-1λ^6^,2-benzothia­zine-1,1,4-trione

**DOI:** 10.1107/S1600536812009002

**Published:** 2012-03-07

**Authors:** Hamid Latif Siddiqui, Matloob Ahmad, Salman Gul, Waseeq Ahmad Siddiqui, Masood Parvez

**Affiliations:** a Institute of Chemistry, University of the Punjab, Lahore 54590, Pakistan; b Chemistry Department, Govt. College University, Faisalabad, Pakistan; c Chemistry Department, University of Sargodha, Sargodha 40100, Pakistan; d Department of Chemistry, The University of Calgary, 2500 University Drive NW, Calgary, Alberta, Canada T2N 1N4

## Abstract

In the title mol­ecule, C_24_H_19_NO_6_S, the heterocyclic thia­zine ring adopts a half-chair conformation with the S and N atoms displaced by 0.180 (5) and 0.497 (5) Å, respectively, on opposite sides of the mean plane formed by the remaining ring atoms. The benzene rings of the benzothia­zine unit and the meth­oxy­phenyl group are almost coplanar, with the dihedral angle between the mean planes of these rings being 5.9 (2)°, while the benzene ring of the 2-oxo-2-phenyl­ethyl group is inclined at 79.68 (11) and 81.01 (10)°, respectively, to these rings. The mol­ecular structure is consolidated by intra­molecular O—H⋯O and C—H⋯N inter­actions, and the crystal packing is stabilized by weak C—H⋯O hydrogen bonds.

## Related literature

For background information on the synthesis of related compounds, see: Siddiqui *et al.* (2007 ▶). For the biological activity of 1,2-benzothia­zine derivatives, see: Lombardino & Wiseman (1972 ▶); Gupta *et al.* (1993 ▶, 2002 ▶); Zia-ur-Rehman *et al.* (2006 ▶); Ahmad *et al.* (2010 ▶). For a related structure, see: Siddiqui *et al.* (2008 ▶).
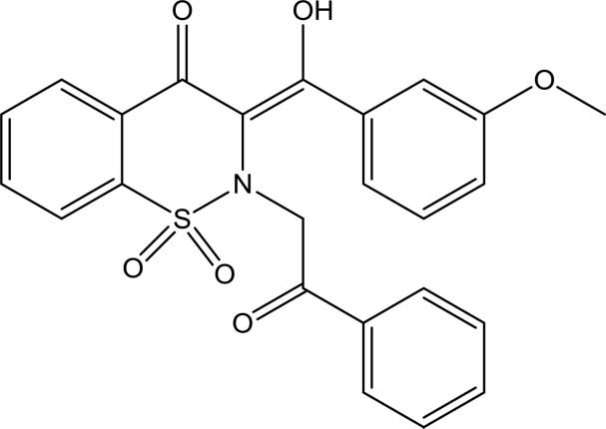


## Experimental

### 

#### Crystal data

C_24_H_19_NO_6_S
*M* *_r_* = 449.46
Orthorhombic, 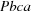

*a* = 17.9615 (5) Å
*b* = 11.2633 (3) Å
*c* = 19.5904 (6) Å
*V* = 3963.3 (2) Å^3^
*Z* = 8
Mo *K*α radiation
μ = 0.21 mm^−1^
*T* = 173 K
0.14 × 0.10 × 0.08 mm

#### Data collection

Nonius KappaCCD diffractometer
Absorption correction: multi-scan (*SORTAV*; Blessing, 1997 ▶) *T*_min_ = 0.971, *T*_max_ = 0.984
8340 measured reflections
4543 independent reflections
3198 reflections with *I* > 2σ(*I*)
*R*_int_ = 0.059

#### Refinement

*R*[*F* ^2^ > 2σ(*F* ^2^)] = 0.068
*wR*(*F* ^2^) = 0.133
*S* = 1.10
4543 reflections
291 parameters
H-atom parameters constrained
Δρ_max_ = 0.36 e Å^−3^
Δρ_min_ = −0.49 e Å^−3^

### 

Data collection: *COLLECT* (Hooft, 1998 ▶); cell refinement: *DENZO* (Otwinowski & Minor, 1997 ▶); data reduction: *SCALEPACK* (Otwinowski & Minor, 1997 ▶); program(s) used to solve structure: *SHELXS97* (Sheldrick, 2008 ▶); program(s) used to refine structure: *SHELXL97* (Sheldrick, 2008 ▶); molecular graphics: *ORTEP-3 for Windows* (Farrugia, 1997 ▶); software used to prepare material for publication: *SHELXL97*.

## Supplementary Material

Crystal structure: contains datablock(s) global, I. DOI: 10.1107/S1600536812009002/pk2393sup1.cif

Structure factors: contains datablock(s) I. DOI: 10.1107/S1600536812009002/pk2393Isup2.hkl

Supplementary material file. DOI: 10.1107/S1600536812009002/pk2393Isup3.cml

Additional supplementary materials:  crystallographic information; 3D view; checkCIF report

## Figures and Tables

**Table 1:** Hydrogen-bond geometry (Å, °)

*D*—H⋯*A*	*D*—H	H⋯*A*	*D*⋯*A*	*D*—H⋯*A*
C21—H21⋯O1^i^	0.95	2.59	3.281 (4)	130
C11—H11⋯O1^ii^	0.95	2.53	3.371 (4)	148
C16—H16*B*⋯O6^iii^	0.98	2.64	3.246 (4)	120
C24—H24⋯O5^iv^	0.95	2.57	3.508 (4)	167
O4—H4*O*⋯O3	0.84	1.71	2.478 (3)	151
C15—H15⋯N1	0.95	2.38	2.972 (4)	120

Symmetry codes: (i) 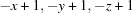; (ii) 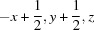; (iii) 
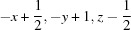
; (iv) 
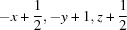
.

**Fig. 1.**
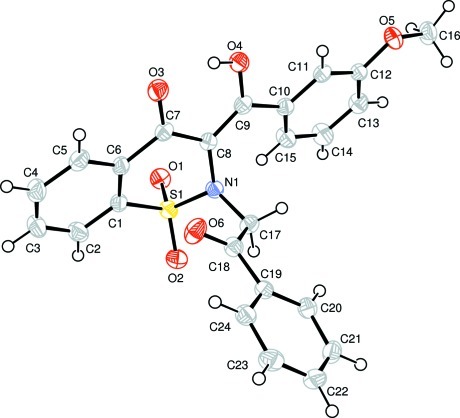
The molecular structure of the title compound. Displacement ellipsoids are drawn at the 50% probability level. H atoms are presented as small spheres of arbitrary radius.

**Fig. 2.**
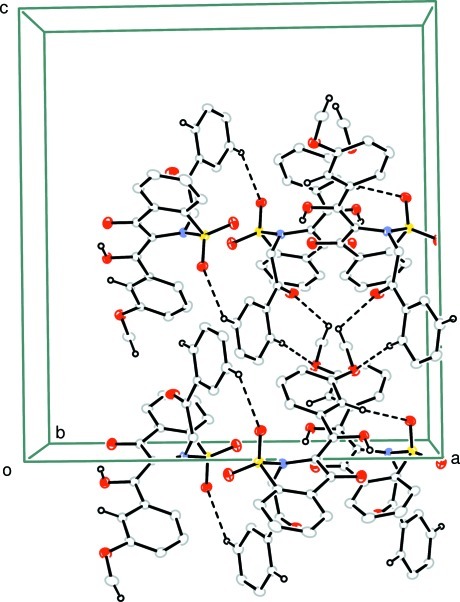
A view of the C—-H···O hydrogen bonds (dotted lines) in the crystal structure of the title compound. H atoms not participating in hydrogen- bonding were omitted for clarity.

## supplementary crystallographic information

### Comment

The derivatives of 1,2-benzothiazine exhibit a wide range of biological activities, *e.g.*, anti inflammatory (Lombardino & Wiseman, 1972), analgesic (Gupta *et al.*, 2002), anti cancer (Gupta *et al.*, 1993) and anti bacterial (Zia-ur-Rehman *et al.*, 2006), *etc*. In continuation of our research on the synthesis of biologically active benzothiazine derivatives (Siddiqui *et al.*, 2007; Ahmad *et al.*, 2010), we herein report the synthesis and crystal structure of the title compound.

The bond distances and angles in the title compound (Fig. 1) agree very well with the corresponding bond distances and angles reported in closely related compounds (Siddiqui *et al.*, 2008). The heterocyclic thiazine ring adopts a half chair conformation with atoms N1 and S1 displaced by 0.497 (6) and 0.180 (6) Å, respectively, on the opposite sides from the mean plane formed by the remaining ring atoms. The benzene rings C1–C6 and C10–C15 are almost co-planar with a dihedral angle between the mean planes of these rings being 5.9 (2)°; the benzene ring C19–C24 is oriented at 79.68 (11) and 81.01 (10)°, respectively, with respect to these benzene rings. While the molecular structure of the title compound is consolidated by intramolecular interactions: O4–H4O···O3 and C15–H15···N1, the crystal packing is stabilized by weak intermolecular C—H···O hydrogen bonds (Fig. 2 and Table 1).

### Experimental

A mixture of (4-hydroxy-1,1-dioxido-2*H*-1,2-benzothiazin-3-yl)(3-methoxyphenyl) methanone (5.0 g, 0.015 mol), K_2_CO_3_ (2.07 g, 0.015 mol) and phenacyl bromide (2.99 g, 0.015 mol) in acetonitrile (30 ml) was refluxed for 3 h. The contents of the flask were poured on ice cold HCl (5%, 30 ml). The precipitates of the title compound formed were collected and washed with ethanol. The crystals suitable for X-ray crystallographic analysis were grown from a solution of methanol.

### Refinement

All H atoms were positioned geometrically and refined using a riding model, with O—H = 0.84 Å and C—H = 0.95, 0.98 and 0.99 Å, for aryl, methyl and methylene H-atoms, respectively. The *U*_iso_(H) were allowed at 1.5*U*_eq_(O) or 1.2*U*_eq_(C).

### Crystal data

**Table:**

C_24_H_19_NO_6_S	*F*(000) = 1872
*M**_r_* = 449.46	*D*_x_ = 1.507 Mg m^−^^3^
Orthorhombic, *P**b**c**a*	Mo *K*α radiation, λ = 0.71073 Å
Hall symbol: -P 2ac 2ab	Cell parameters from 5006 reflections
*a* = 17.9615 (5) Å	θ = 1.0–27.5°
*b* = 11.2633 (3) Å	µ = 0.21 mm^−^^1^
*c* = 19.5904 (6) Å	*T* = 173 K
*V* = 3963.3 (2) Å^3^	Prism, yellow
*Z* = 8	0.14 × 0.10 × 0.08 mm

### Data collection

**Table:**

Nonius KappaCCD diffractometer	4543 independent reflections
Radiation source: fine-focus sealed tube	3198 reflections with *I* > 2σ(*I*)
Graphite monochromator	*R*_int_ = 0.059
ω and φ scans	θ_max_ = 27.5°, θ_min_ = 3.0°
Absorption correction: multi-scan (*SORTAV*; Blessing, 1997)	*h* = −23→23
*T*_min_ = 0.971, *T*_max_ = 0.984	*k* = −14→14
8340 measured reflections	*l* = −25→25

### Refinement

**Table:**

Refinement on *F*^2^	Primary atom site location: structure-invariant direct methods
Least-squares matrix: full	Secondary atom site location: difference Fourier map
*R*[*F*^2^ > 2σ(*F*^2^)] = 0.068	Hydrogen site location: inferred from neighbouring sites
*wR*(*F*^2^) = 0.133	H-atom parameters constrained
*S* = 1.10	*w* = 1/[σ^2^(*F*_o_^2^) + 10.1913*P*] where *P* = (*F*_o_^2^ + 2*F*_c_^2^)/3
4543 reflections	(Δ/σ)_max_ < 0.001
291 parameters	Δρ_max_ = 0.36 e Å^−^^3^
0 restraints	Δρ_min_ = −0.49 e Å^−^^3^

### Special details

**Table:**

Geometry. All e.s.d.'s (except the e.s.d. in the dihedral angle between two l.s. planes) are estimated using the full covariance matrix. The cell e.s.d.'s are taken into account individually in the estimation of e.s.d.'s in distances, angles and torsion angles; correlations between e.s.d.'s in cell parameters are only used when they are defined by crystal symmetry. An approximate (isotropic) treatment of cell e.s.d.'s is used for estimating e.s.d.'s involving l.s. planes.
Refinement. Refinement of *F*^2^ against ALL reflections. The weighted *R*-factor *wR* and goodness of fit *S* are based on *F*^2^, conventional *R*-factors *R* are based on *F*, with *F* set to zero for negative *F*^2^. The threshold expression of *F*^2^ > σ(*F*^2^) is used only for calculating *R*-factors(gt) *etc*. and is not relevant to the choice of reflections for refinement. *R*-factors based on *F*^2^ are statistically about twice as large as those based on *F*, and *R*- factors based on ALL data will be even larger.

### Fractional atomic coordinates and isotropic or equivalent isotropic displacement parameters (Å^2^)

**Table:**

	*x*	*y*	*z*	*U*_iso_*/*U*_eq_	
S1	0.43448 (4)	0.23066 (7)	0.49448 (4)	0.02772 (19)	
O1	0.42975 (13)	0.1891 (2)	0.42537 (11)	0.0321 (5)	
O2	0.50593 (12)	0.2608 (2)	0.52165 (13)	0.0380 (6)	
O3	0.19812 (12)	0.2130 (2)	0.52567 (12)	0.0334 (6)	
O4	0.18789 (12)	0.3846 (2)	0.44712 (12)	0.0311 (5)	
H4O	0.1754	0.3308	0.4745	0.047*	
O5	0.20407 (13)	0.7401 (2)	0.31274 (12)	0.0350 (6)	
O6	0.35370 (14)	0.3374 (2)	0.64057 (12)	0.0395 (6)	
N1	0.38071 (14)	0.3469 (2)	0.50267 (13)	0.0248 (6)	
C1	0.39090 (18)	0.1240 (3)	0.54646 (16)	0.0259 (7)	
C2	0.4322 (2)	0.0323 (3)	0.57373 (17)	0.0358 (8)	
H2	0.4841	0.0268	0.5650	0.043*	
C3	0.3971 (2)	−0.0515 (3)	0.6140 (2)	0.0421 (9)	
H3	0.4248	−0.1157	0.6325	0.051*	
C4	0.3218 (2)	−0.0425 (3)	0.62739 (18)	0.0399 (9)	
H4	0.2980	−0.0998	0.6555	0.048*	
C5	0.2809 (2)	0.0502 (3)	0.59989 (17)	0.0325 (8)	
H5	0.2292	0.0561	0.6096	0.039*	
C6	0.31417 (18)	0.1342 (3)	0.55844 (16)	0.0264 (7)	
C7	0.26845 (17)	0.2284 (3)	0.52620 (16)	0.0261 (7)	
C8	0.30240 (16)	0.3262 (3)	0.49250 (16)	0.0231 (6)	
C9	0.26011 (18)	0.3991 (3)	0.44929 (16)	0.0262 (7)	
C10	0.28801 (17)	0.4937 (3)	0.40321 (16)	0.0256 (7)	
C11	0.23700 (17)	0.5772 (3)	0.37964 (15)	0.0254 (7)	
H11	0.1871	0.5756	0.3958	0.030*	
C12	0.25855 (18)	0.6626 (3)	0.33282 (16)	0.0271 (7)	
C13	0.33096 (19)	0.6660 (3)	0.30894 (17)	0.0331 (8)	
H13	0.3459	0.7251	0.2772	0.040*	
C14	0.3811 (2)	0.5824 (3)	0.33196 (19)	0.0386 (9)	
H14	0.4307	0.5839	0.3152	0.046*	
C15	0.36098 (19)	0.4962 (3)	0.37892 (18)	0.0343 (8)	
H15	0.3964	0.4397	0.3944	0.041*	
C16	0.2218 (2)	0.8229 (3)	0.25964 (18)	0.0384 (9)	
H16A	0.1770	0.8679	0.2473	0.046*	
H16B	0.2400	0.7797	0.2195	0.046*	
H16C	0.2604	0.8776	0.2757	0.046*	
C17	0.40483 (18)	0.4447 (3)	0.54746 (16)	0.0280 (7)	
H17A	0.3794	0.5185	0.5330	0.034*	
H17B	0.4590	0.4568	0.5414	0.034*	
C18	0.38901 (17)	0.4240 (3)	0.62268 (17)	0.0269 (7)	
C19	0.41875 (17)	0.5095 (3)	0.67386 (17)	0.0258 (7)	
C20	0.45909 (18)	0.6100 (3)	0.65584 (17)	0.0286 (7)	
H20	0.4641	0.6311	0.6091	0.034*	
C21	0.49201 (19)	0.6796 (3)	0.70580 (18)	0.0334 (8)	
H21	0.5206	0.7471	0.6932	0.040*	
C22	0.4834 (2)	0.6511 (3)	0.77347 (18)	0.0359 (8)	
H22	0.5068	0.6981	0.8075	0.043*	
C23	0.4404 (2)	0.5534 (3)	0.79248 (18)	0.0362 (8)	
H23	0.4326	0.5359	0.8394	0.043*	
C24	0.40910 (18)	0.4825 (3)	0.74264 (17)	0.0299 (7)	
H24	0.3808	0.4148	0.7554	0.036*	

### Atomic displacement parameters (Å^2^)

**Table:**

	*U*^11^	*U*^22^	*U*^33^	*U*^12^	*U*^13^	*U*^23^
S1	0.0271 (4)	0.0275 (4)	0.0285 (4)	0.0017 (3)	−0.0005 (3)	0.0012 (3)
O1	0.0348 (12)	0.0336 (12)	0.0277 (12)	0.0028 (11)	0.0035 (10)	−0.0028 (10)
O2	0.0287 (12)	0.0381 (13)	0.0473 (15)	0.0006 (11)	−0.0057 (11)	0.0028 (12)
O3	0.0288 (12)	0.0310 (12)	0.0403 (14)	−0.0025 (10)	0.0033 (10)	0.0038 (11)
O4	0.0265 (12)	0.0304 (12)	0.0364 (14)	−0.0029 (10)	0.0013 (10)	0.0082 (11)
O5	0.0405 (13)	0.0301 (12)	0.0344 (13)	0.0050 (11)	−0.0015 (11)	0.0086 (11)
O6	0.0571 (16)	0.0347 (13)	0.0266 (13)	−0.0163 (12)	0.0024 (12)	0.0024 (11)
N1	0.0243 (13)	0.0251 (13)	0.0251 (14)	0.0001 (11)	−0.0001 (11)	0.0012 (11)
C1	0.0338 (17)	0.0226 (15)	0.0215 (16)	−0.0026 (13)	−0.0043 (14)	0.0000 (13)
C2	0.044 (2)	0.0330 (18)	0.0306 (19)	0.0050 (17)	−0.0093 (16)	−0.0002 (15)
C3	0.055 (2)	0.0300 (19)	0.041 (2)	0.0033 (18)	−0.0148 (19)	0.0066 (17)
C4	0.060 (2)	0.0301 (18)	0.0298 (19)	−0.0086 (18)	−0.0089 (18)	0.0080 (16)
C5	0.0401 (19)	0.0301 (17)	0.0272 (18)	−0.0087 (15)	−0.0016 (15)	−0.0010 (14)
C6	0.0362 (18)	0.0205 (15)	0.0225 (16)	−0.0017 (13)	−0.0057 (14)	−0.0018 (13)
C7	0.0269 (16)	0.0242 (15)	0.0271 (17)	−0.0025 (13)	0.0007 (13)	−0.0045 (13)
C8	0.0244 (15)	0.0232 (14)	0.0217 (16)	−0.0002 (12)	0.0008 (13)	−0.0006 (13)
C9	0.0290 (17)	0.0252 (15)	0.0245 (16)	−0.0010 (13)	0.0012 (13)	−0.0056 (13)
C10	0.0298 (17)	0.0243 (15)	0.0226 (16)	−0.0006 (13)	−0.0020 (13)	−0.0016 (13)
C11	0.0271 (16)	0.0255 (15)	0.0234 (16)	−0.0030 (13)	−0.0001 (13)	−0.0045 (13)
C12	0.0315 (17)	0.0230 (15)	0.0268 (16)	0.0015 (14)	−0.0067 (14)	−0.0028 (13)
C13	0.0372 (19)	0.0336 (18)	0.0284 (18)	−0.0043 (16)	0.0039 (15)	0.0054 (15)
C14	0.0329 (18)	0.042 (2)	0.041 (2)	0.0014 (16)	0.0088 (17)	0.0093 (17)
C15	0.0319 (18)	0.0355 (18)	0.036 (2)	0.0035 (15)	0.0008 (15)	0.0071 (16)
C16	0.055 (2)	0.0279 (17)	0.032 (2)	0.0025 (17)	−0.0058 (17)	0.0035 (15)
C17	0.0265 (16)	0.0281 (16)	0.0294 (18)	−0.0049 (14)	0.0008 (14)	0.0001 (14)
C18	0.0262 (16)	0.0272 (16)	0.0274 (17)	−0.0004 (13)	0.0020 (14)	0.0033 (14)
C19	0.0216 (15)	0.0253 (15)	0.0305 (18)	0.0028 (13)	−0.0022 (13)	−0.0005 (13)
C20	0.0309 (17)	0.0276 (16)	0.0271 (17)	0.0010 (14)	0.0016 (14)	0.0027 (14)
C21	0.0349 (18)	0.0244 (16)	0.041 (2)	−0.0004 (14)	−0.0003 (16)	−0.0031 (15)
C22	0.0378 (19)	0.0350 (18)	0.035 (2)	0.0003 (16)	−0.0092 (16)	−0.0066 (16)
C23	0.040 (2)	0.044 (2)	0.0246 (17)	0.0019 (17)	−0.0032 (15)	−0.0015 (15)
C24	0.0279 (16)	0.0306 (16)	0.0312 (18)	0.0000 (14)	−0.0010 (14)	0.0048 (15)

### Geometric parameters (Å, º)

**Table:**

S1—O2	1.430 (2)	C10—C15	1.395 (4)
S1—O1	1.435 (2)	C11—C12	1.385 (4)
S1—N1	1.635 (3)	C11—H11	0.9500
S1—C1	1.759 (3)	C12—C13	1.383 (5)
O3—C7	1.275 (4)	C13—C14	1.379 (5)
O4—C9	1.308 (4)	C13—H13	0.9500
O4—H4O	0.8400	C14—C15	1.385 (5)
O5—C12	1.369 (4)	C14—H14	0.9500
O5—C16	1.432 (4)	C15—H15	0.9500
O6—C18	1.215 (4)	C16—H16A	0.9800
N1—C8	1.440 (4)	C16—H16B	0.9800
N1—C17	1.473 (4)	C16—H16C	0.9800
C1—C2	1.380 (4)	C17—C18	1.519 (4)
C1—C6	1.403 (4)	C17—H17A	0.9900
C2—C3	1.383 (5)	C17—H17B	0.9900
C2—H2	0.9500	C18—C19	1.489 (4)
C3—C4	1.381 (5)	C19—C20	1.389 (4)
C3—H3	0.9500	C19—C24	1.392 (4)
C4—C5	1.385 (5)	C20—C21	1.386 (5)
C4—H4	0.9500	C20—H20	0.9500
C5—C6	1.383 (4)	C21—C22	1.373 (5)
C5—H5	0.9500	C21—H21	0.9500
C6—C7	1.483 (4)	C22—C23	1.395 (5)
C7—C8	1.421 (4)	C22—H22	0.9500
C8—C9	1.403 (4)	C23—C24	1.381 (5)
C9—C10	1.483 (4)	C23—H23	0.9500
C10—C11	1.392 (4)	C24—H24	0.9500
			
O2—S1—O1	118.80 (15)	O5—C12—C11	115.7 (3)
O2—S1—N1	107.66 (14)	C13—C12—C11	120.4 (3)
O1—S1—N1	108.60 (14)	C14—C13—C12	119.0 (3)
O2—S1—C1	110.25 (15)	C14—C13—H13	120.5
O1—S1—C1	107.29 (14)	C12—C13—H13	120.5
N1—S1—C1	103.14 (14)	C13—C14—C15	121.7 (3)
C9—O4—H4O	109.5	C13—C14—H14	119.2
C12—O5—C16	117.7 (3)	C15—C14—H14	119.2
C8—N1—C17	119.4 (2)	C14—C15—C10	119.1 (3)
C8—N1—S1	115.7 (2)	C14—C15—H15	120.4
C17—N1—S1	118.9 (2)	C10—C15—H15	120.4
C2—C1—C6	121.7 (3)	O5—C16—H16A	109.5
C2—C1—S1	119.7 (3)	O5—C16—H16B	109.5
C6—C1—S1	118.6 (2)	H16A—C16—H16B	109.5
C1—C2—C3	119.1 (3)	O5—C16—H16C	109.5
C1—C2—H2	120.4	H16A—C16—H16C	109.5
C3—C2—H2	120.4	H16B—C16—H16C	109.5
C4—C3—C2	120.3 (3)	N1—C17—C18	114.1 (3)
C4—C3—H3	119.8	N1—C17—H17A	108.7
C2—C3—H3	119.8	C18—C17—H17A	108.7
C3—C4—C5	120.1 (3)	N1—C17—H17B	108.7
C3—C4—H4	120.0	C18—C17—H17B	108.7
C5—C4—H4	120.0	H17A—C17—H17B	107.6
C6—C5—C4	121.0 (3)	O6—C18—C19	120.8 (3)
C6—C5—H5	119.5	O6—C18—C17	120.0 (3)
C4—C5—H5	119.5	C19—C18—C17	119.2 (3)
C5—C6—C1	117.8 (3)	C20—C19—C24	119.3 (3)
C5—C6—C7	120.0 (3)	C20—C19—C18	122.9 (3)
C1—C6—C7	122.1 (3)	C24—C19—C18	117.7 (3)
O3—C7—C8	121.8 (3)	C21—C20—C19	120.2 (3)
O3—C7—C6	117.0 (3)	C21—C20—H20	119.9
C8—C7—C6	121.0 (3)	C19—C20—H20	119.9
C9—C8—C7	120.1 (3)	C22—C21—C20	120.1 (3)
C9—C8—N1	121.2 (3)	C22—C21—H21	120.0
C7—C8—N1	118.7 (3)	C20—C21—H21	120.0
O4—C9—C8	118.9 (3)	C21—C22—C23	120.3 (3)
O4—C9—C10	113.9 (3)	C21—C22—H22	119.8
C8—C9—C10	127.2 (3)	C23—C22—H22	119.8
C11—C10—C15	119.4 (3)	C24—C23—C22	119.5 (3)
C11—C10—C9	117.7 (3)	C24—C23—H23	120.2
C15—C10—C9	122.7 (3)	C22—C23—H23	120.2
C12—C11—C10	120.4 (3)	C23—C24—C19	120.5 (3)
C12—C11—H11	119.8	C23—C24—H24	119.8
C10—C11—H11	119.8	C19—C24—H24	119.8
O5—C12—C13	123.9 (3)		
			
O2—S1—N1—C8	−166.6 (2)	N1—C8—C9—O4	−172.9 (3)
O1—S1—N1—C8	63.6 (2)	C7—C8—C9—C10	−171.0 (3)
C1—S1—N1—C8	−50.0 (3)	N1—C8—C9—C10	7.8 (5)
O2—S1—N1—C17	−13.9 (3)	O4—C9—C10—C11	18.6 (4)
O1—S1—N1—C17	−143.8 (2)	C8—C9—C10—C11	−162.1 (3)
C1—S1—N1—C17	102.6 (2)	O4—C9—C10—C15	−156.3 (3)
O2—S1—C1—C2	−40.4 (3)	C8—C9—C10—C15	23.0 (5)
O1—S1—C1—C2	90.4 (3)	C15—C10—C11—C12	−0.4 (5)
N1—S1—C1—C2	−155.1 (3)	C9—C10—C11—C12	−175.5 (3)
O2—S1—C1—C6	140.3 (2)	C16—O5—C12—C13	5.5 (5)
O1—S1—C1—C6	−88.9 (3)	C16—O5—C12—C11	−174.2 (3)
N1—S1—C1—C6	25.6 (3)	C10—C11—C12—O5	179.7 (3)
C6—C1—C2—C3	−0.1 (5)	C10—C11—C12—C13	0.0 (5)
S1—C1—C2—C3	−179.4 (3)	O5—C12—C13—C14	−179.1 (3)
C1—C2—C3—C4	−0.9 (5)	C11—C12—C13—C14	0.6 (5)
C2—C3—C4—C5	0.7 (6)	C12—C13—C14—C15	−0.8 (6)
C3—C4—C5—C6	0.5 (5)	C13—C14—C15—C10	0.4 (6)
C4—C5—C6—C1	−1.4 (5)	C11—C10—C15—C14	0.2 (5)
C4—C5—C6—C7	176.3 (3)	C9—C10—C15—C14	175.1 (3)
C2—C1—C6—C5	1.2 (5)	C8—N1—C17—C18	69.8 (4)
S1—C1—C6—C5	−179.5 (2)	S1—N1—C17—C18	−81.8 (3)
C2—C1—C6—C7	−176.4 (3)	N1—C17—C18—O6	−5.8 (4)
S1—C1—C6—C7	2.9 (4)	N1—C17—C18—C19	172.8 (3)
C5—C6—C7—O3	−15.2 (4)	O6—C18—C19—C20	−179.7 (3)
C1—C6—C7—O3	162.4 (3)	C17—C18—C19—C20	1.7 (5)
C5—C6—C7—C8	169.9 (3)	O6—C18—C19—C24	4.3 (5)
C1—C6—C7—C8	−12.5 (5)	C17—C18—C19—C24	−174.3 (3)
O3—C7—C8—C9	−9.3 (5)	C24—C19—C20—C21	2.7 (5)
C6—C7—C8—C9	165.3 (3)	C18—C19—C20—C21	−173.2 (3)
O3—C7—C8—N1	171.9 (3)	C19—C20—C21—C22	−1.5 (5)
C6—C7—C8—N1	−13.5 (4)	C20—C21—C22—C23	−1.3 (5)
C17—N1—C8—C9	76.6 (4)	C21—C22—C23—C24	2.8 (5)
S1—N1—C8—C9	−130.9 (3)	C22—C23—C24—C19	−1.6 (5)
C17—N1—C8—C7	−104.6 (3)	C20—C19—C24—C23	−1.2 (5)
S1—N1—C8—C7	47.9 (3)	C18—C19—C24—C23	175.0 (3)
C7—C8—C9—O4	8.2 (4)		

### Hydrogen-bond geometry (Å, º)

**Table:**

*D*—H···*A*	*D*—H	H···*A*	*D*···*A*	*D*—H···*A*
C21—H21···O1^i^	0.95	2.59	3.281 (4)	130
C11—H11···O1^ii^	0.95	2.53	3.371 (4)	148
C16—H16*B*···O6^iii^	0.98	2.64	3.246 (4)	120
C24—H24···O5^iv^	0.95	2.57	3.508 (4)	167
O4—H4*O*···O3	0.84	1.71	2.478 (3)	151
C15—H15···N1	0.95	2.38	2.972 (4)	120
C17—H17*B*···O2	0.99	2.39	2.800 (4)	104

Symmetry codes: (i) −*x*+1, −*y*+1, −*z*+1; (ii) −*x*+1/2, *y*+1/2, *z*; (iii) −*x*+1/2, −*y*+1, *z*−1/2; (iv) −*x*+1/2, −*y*+1, *z*+1/2.

## References

[bb1] Ahmad, M., Siddiqui, H. L., Zia-ur-Rehman, M. & Parvez, M. (2010). *Eur. J. Med. Chem.* **45**, 698–704. 10.1016/j.ejmech.2009.11.016 19962218

[bb2] Blessing, R. H. (1997). *J. Appl. Cryst.* **30**, 421–426.

[bb3] Farrugia, L. J. (1997). *J. Appl. Cryst.* **30**, 565.

[bb4] Gupta, S. K., Bansal, P., Bhardwaj, R. K., Jaiswal, J. & Velpandian, T. (2002). *Skin Pharmacol. Appl. Skin Physiol.* **15**, 105–111. 10.1159/000049397 11867967

[bb5] Gupta, R. R., Dev, P. K., Sharma, M. L., Rajoria, C. M., Gupta, A. & Nyati, M. (1993). *Anticancer Drugs*, **4**, 589–592. 10.1097/00001813-199310000-00010 8292818

[bb6] Hooft, R. (1998). *COLLECT* Nonius BV, Delft, The Netherlands.

[bb7] Lombardino, J. G. & Wiseman, E. H. (1972). *J. Med. Chem.* **15**, 848–849. 10.1021/jm00278a016 4625532

[bb8] Otwinowski, Z. & Minor, W. (1997). *Methods in Enzymology*, Vol. 276, *Macromolecular Crystallography*, Part A, edited by C. W. Carter Jr & R. M. Sweet, pp. 307–326. New York: Academic Press.

[bb9] Sheldrick, G. M. (2008). *Acta Cryst.* A**64**, 112–122. 10.1107/S0108767307043930 18156677

[bb10] Siddiqui, W. A., Ahmad, S., Khan, I. U., Siddiqui, H. L. & Weaver, G. W. (2007). *Synth. Commun.* **37**, 767–773.

[bb11] Siddiqui, W. A., Ahmad, S., Tariq, M. I., Siddiqui, H. L. & Parvez, M. (2008). *Acta Cryst.* C**64**, o4–o6. 10.1107/S0108270107059173 18216455

[bb12] Zia-ur-Rehman, M., Choudary, J. A., Ahmad, S. & Siddiqui, H. L. (2006). *Chem. Pharm. Bull.* **54**, 1175–1178. 10.1248/cpb.54.1175 16880664

